# A combined anterior cruciate ligament/Meniscal injury alters the patellofemoral joint kinematics of anterior cruciate ligament-deficient knees during a single-leg lunge exercise: A cross-sectional study

**DOI:** 10.3389/fbioe.2022.1016859

**Published:** 2022-11-03

**Authors:** Wenhan Huang, Xiaolong Zeng, Mengyuan Li, Haobin Chen, Huahao Lai, Yuan Yan, Hua Zhong, Yu Zhang

**Affiliations:** ^1^ Department of Orthopaedics, Guangdong Provincial People’s Hospital, Guangdong Academy of Medical Sciences, Guanzhou, China; ^2^ Department of Orthopaedics, The Fifth Affiliated Hospital, Southern Medical University, Guangzhou, China

**Keywords:** anterior cruciate ligament deficiency, meniscal injury, patellofemoral joint kinematics, single-leg lunge, knee

## Abstract

Anterior cruciate ligament deficiency (ACLD) is often accompanied by concomitant meniscal tears. The study aimed to assess the kinematic alterations of patellofemoral joint (PFJ) in anterior cruciate ligament deficiency knees with or without meniscal tears during a single-leg lunge. Sixty unilateral anterior cruciate ligament deficiency patients were recruited for the study, including 15 isolated anterior cruciate ligament deficiency patients (group 1), 15 anterior cruciate ligament deficiency patients with medial meniscal tears (group 2), 15 patients with lateral meniscal tears (group 3) and 15 patients with combined medial/lateral meniscal tears (group 4). The patellofemoral joint kinematics were determined by a single fluoroscopic image system. Patellofemoral joint kinematics of contralateral anterior cruciate ligament-intact (ACLI) and anterior cruciate ligament deficiency knees were compared. With or without meniscal tears, anterior cruciate ligament deficiency knees had significantly smaller patellar flexion than the anterior cruciate ligament-intact knees (∼5°–10°; *p* < 0.05). anterior cruciate ligament deficiency knees had more patellar lateral tilting by approximately 1°–2° than the anterior cruciate ligament-intact knees (*p* < 0.05) in groups 2, 3, and 4. anterior cruciate ligament deficiency groups with medial meniscal deficiencies showed consistent increased lateral patellar translations (2–4 mm) compared to the anterior cruciate ligament-intact group during a single-leg lunge. The results indicate that meniscal tears alter anterior cruciate ligament deficiency patients’ patellofemoral joint kinematics and the types of the meniscal injuries also affect the patellofemoral joint kinematics. Considering the varying effects of meniscal tears on the patellofemoral joint kinematics, specific treatments for anterior cruciate ligament deficiency patients with meniscal tears should be proposed in some closed kinetic chain (CKC) exercise programs, such as single-leg lunge.

## 1 Introduction

Anterior cruciate ligament (ACL) injuries, which account for more than 40% of sports injuries, are the most common sport-related trauma (Gordon, 2004). Numerous researchers have investigated the tibiofemoral joint (TFJ) biomechanics in ACL-deficient (ACLD) knees with or without concomitant meniscus deficiency but ignored patellofemoral joint (PFJ) biomechanics. ACL ruptures are commonly combined with meniscal injuries. A meta-analysis by Bellabarba et al. reported that 41–82% of acute ACLD knees and 58–100% of chronic ACLD knees had meniscal tears (Bellabarba et al., 1997). Lin reported that ACLD knees had reduced distal patellar translation and patellar flexion angle during knee flexion (Lin et al., 2019). Although ACLD is known to lead to abnormal anteroposterior tibial motions, the influence of combined meniscal tears on the PFJ functionality is not clear, especially during daily movements like walking, jogging, lunging, and stair descending/ascending.

A lunge is a single-leg body-weight exercise that involves our hips, gluteus, quadriceps, hamstrings, core, and the hard-to-reach muscles of our inner thighs. Lunges, as a kind of closed kinetic chain (CKC) exercises, have been preferentially used due to the belief that it is safe for replicated functional tasks and does not increase the risk of anterior tibial displacement (Perry et al., 2005; Perry et al., 2005). The ACL has an essential role in stabilizing knee joint by not only maintaining anterior tibial translation but also limiting the axial and transverse rotation of the knee. Therefore, many athletes who accepted nonsurgical treatment after ACL injury perform lunge exercises because, when done correctly, lunges can effectively target the athlete’s quadriceps muscle group without placing added strain on the knee joints (Tagesson et al., 2008). In 2015, researchers found that swimmers had faster swimming times after warming up with either lunges or squats (Cuenca-Fernandez et al., 2015). However, whether doing lunges are detrimental to the PFJ remains unknown. Culvenor et al. reported that PFJ cartilage thickness loss of young adults was found following acute ACLD (Culvenor et al., 2019). Therefore, for those who accepted delayed ACLR or rehabilitation alone, it is essential to understand the effect of the rehabilitation program on the PFJ biomechanical environment.

The patella is an important component of the knee. Because the patellar tendon attaches the tibial tubercle, the movement of the tibia is believed to affect patellar movements. Lee et al. reported that the patella’s distal attachment to the tibial tubercle has an effect on the directions of patellar movement (Lee et al., 2003). During external tibial rotation, the patellar tendon functions by laterally pulling on the distal pole of the patella, thus rotating the superior aspect of the patella medially. Therefore, logically, any kinematic changes that result in alterations to the tibia or patellar positions relative to the femur during ACL deficiency are likely to be associated with PFJ disorders. However, there is no direct evidence to support this theory. When the contact locations between the patella and the femur are altered, abnormal distribution of the stresses on the PFJ occurs. This abnormal distribution of stresses is considered to be strongly correlated to adverse PFJ outcomes, such as chondromalacia and subsequent osteoarthritis (OA) (Hvid et al., 1981). Previous studies have demonstrated that ACLD alter the knee kinematics with or without meniscal tears. However, whether the alterations of knee joint kinematics affect the PFJ kinematics remains unknown.

Hence, the present study was to determine the PFJ kinematics after an ACLD with or without a combined medial or lateral meniscal rupture. A single fluoroscopic image system was used to determine dynamic patella movement and determine the 6 degrees of freedom (DOF) knee kinematics of the ACLD knees and the ACL-intact (ACLI) knees during a single-leg lunge exercise. We hypothesized that the ACLD patients’ PFJ kinematics varies based on meniscal rupture location (medial, lateral, or medial and lateral) during a single-leg lunge exercise.

## 2 Methods

### 2.1 Participants

60 unilateral ACLD patients with (age range, 20–35 years; body mass index range, 18.1–25.6 kg/m^2^; 38 male and 22 female) were enrolled for the study. These patients were divided into four groups according to the status of meniscus including Group 1 (15 patients, isolated ACL injuries), Group 2 (15 patients, combined ACL and medial meniscal injuries), Group 3 (15 patients, combined ACL and lateral meniscal injuries), and Group 4 (combined ACL and medial/lateral meniscal injuries). These patients were diagnosed with unilateral ACLD *via* magnetic resonance imaging (MRI) and a clinical examination within a year (average 3 months). Regarding with the effects of effusion, the patella tap tests were taken to examine the patients and all patients showed negative results. The type of meniscal tear (i.e., horizontal cleavage tear, posterior meniscal root tear, or radial tear) was not considered because of the limited sample size. A single experienced orthopaedic doctor performed the physical examination and made the MRI diagnosis. Complete ACL ruptures and the locations of the meniscal injuries were confirmed *via* MRI examination. The contralateral (control) knees had no history of knee injuries. This study was approved by an institutional review board [GDREC2019226H(R1)]. Written informed consents were received from all the patients. The description on patient and public involvement was shown in Appendix 1. The technical details were described in Appendix 2.

### 2.2 Creation of 3D model

**FIGURE 1:**
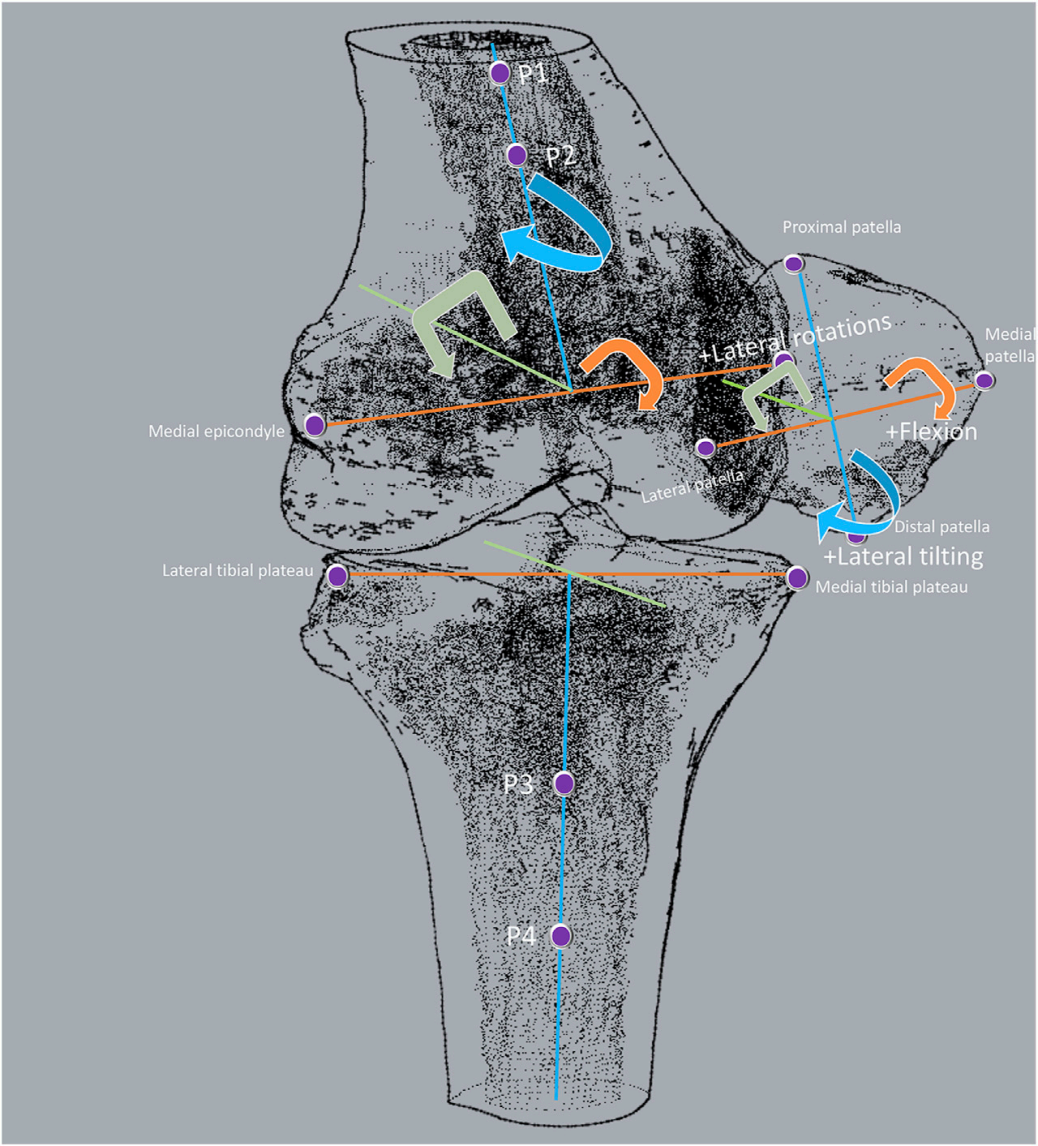
Definition of patellofemoral and tibiofemoral coordinate systems. The “4-points” method was employed to build coordinate systems in the femur, patella, and tibia. In the femur, the first two points were the prominent points of the medial and lateral femoral epicondyles. The other two points formed a line parallel to the sides of the femur shaft. In the patella, the first two points were the most medial and lateral points on the patella. The other two points were the most proximal and distal points on the patella. The line connected to the most medial and lateral points on the patella was defined as the medial-lateral axis, and the midpoint of this line was defined as the origin of the patella. In the tibia, the first two points were the most external points on the sides of the medial and lateral tibia plateau. The other two points formed a line parallel to the sides of the tibia shaft. PFJ kinematics was the movement of patella relative to the middle point of the prominent points of the medial and lateral femoral epicondyles, including patellar flexion/extension, varus/valgus, medial/lateral tilt, anteroposterior translation, medial/lateral translation, and proximal/distal translation. TFJ kinematics was the movement of tibia relative to the middle point of the most external points on the sides of the medial and lateral tibia plateau, including tibial flexion/extension, varus/valgus, medial/lateral rotation, anterior/posterior translation, distal/proximal translation.

The knee joint of each patient was recorded using computed tomography (CT) (SOMATOM Definition; Siemens, Munich, Germany). Parallel digital images with a thickness of 1 mm without a gap and with a resolution of 512*512 pixels were obtained. The images were then imported into solid modeling software Mimics 17.0 (Masterialise NV, Leuven, Belgium) and manually digitized to outline the contours of the femur, the patella, and the tibia. These outlines were used to construct three-dimensional geometric models of the knees. Two consistent coordinate systems (PFJ and TFJ) were used to estimate the 6DOF kinematics of the PFJ and the TFJ in both knees of each participant based on the series of matched bone models (Figure 1).

### 2.3 Determination of *in vivo* patellofemoral joint kinematics kinematics

**FIGURE 2:**
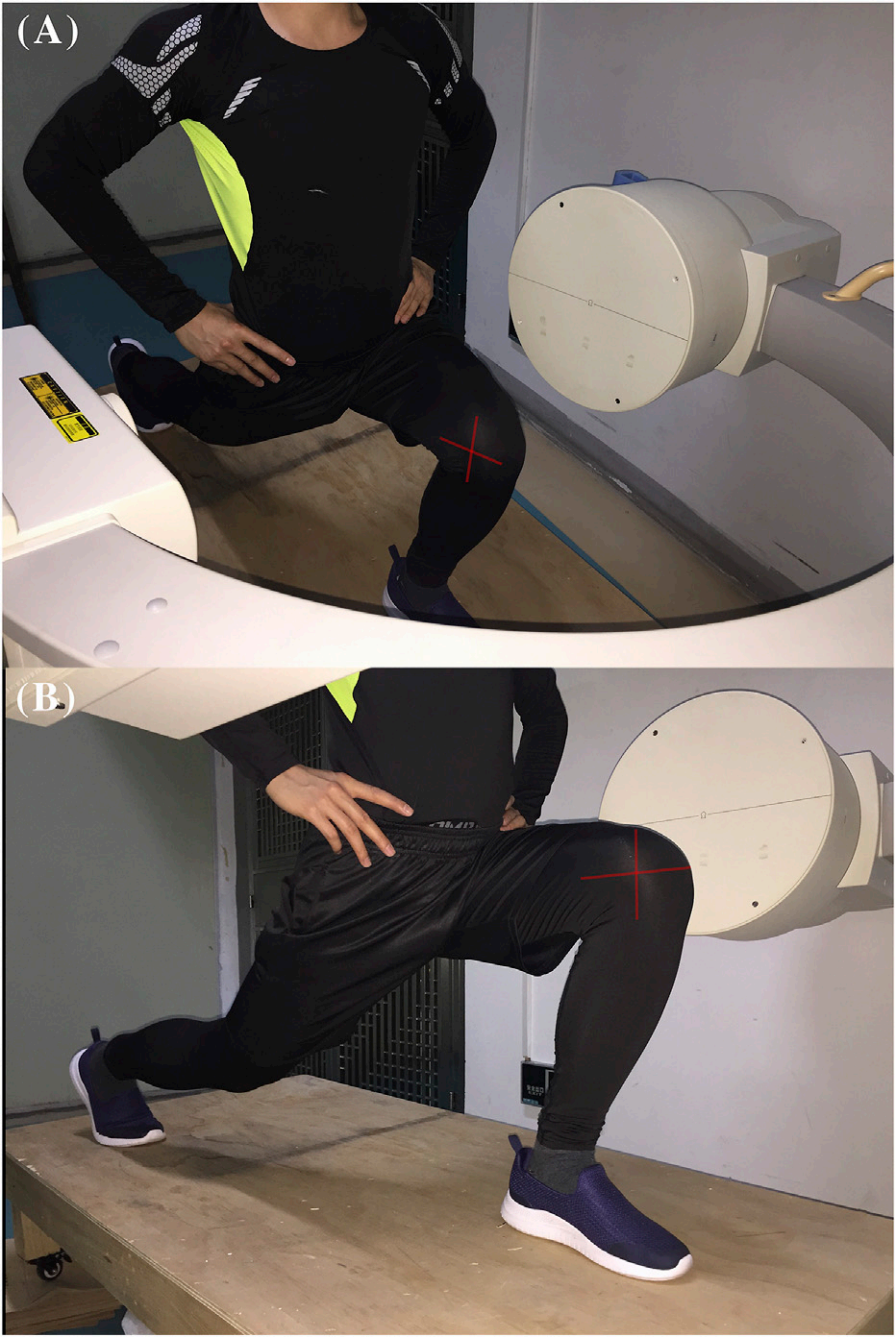
The single fluoroscopic imaging system with a patient performing a single-leg lunge.

**FIGURE 3:**
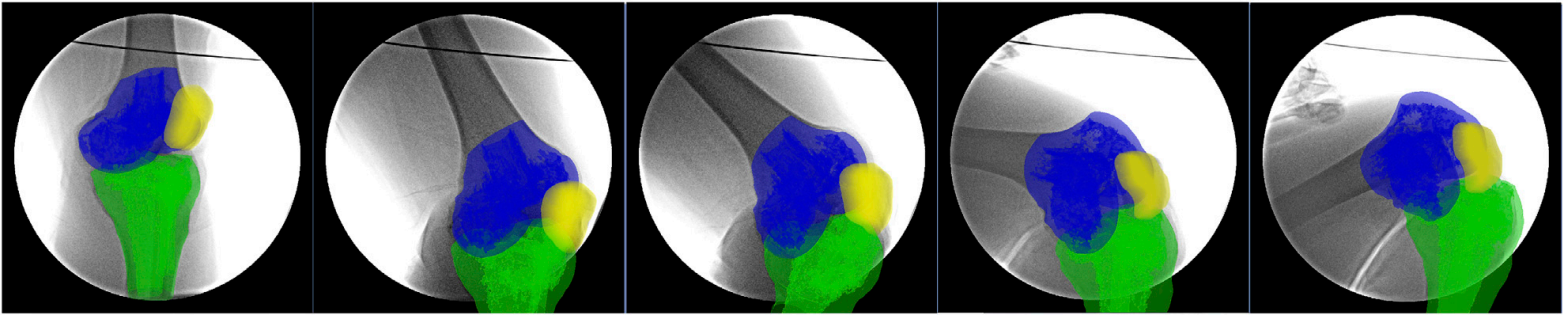
The real-time fluoroscopic images of the knee at a specific posture during single-leg lunge.

A validated single-plane fluoroscopic imaging system was used to determine the PFJ 6DOF kinematics of both the intact knees and injured knees during a single-leg lunge (Van de Velde et al., 2008). Laser-positioning devices, attached to the fluoroscopes, help to align the target knee within the field of view of the fluoroscopes while participants performed a single-leg lunge. Each participant was asked to stand with his or her feet hip-width apart, keep his or her back and shoulders straight, and his or her rectus abdominis muscle tight. Next, each participant took a big step forward and slowly bended both knees until his or her back knee was just above the floor (Figure 2). The other leg was only used to maintain body stability. The knee postures of test participants were carefully examined under the direction of an orthopedic surgeon to reduce variation. No constraint was applied to both knees of the participants while they performed active motions. The entire experiment took approximately 5 min to complete, and images were processed in the Digital Imaging and Communications in Medicine (DICOM) formats (http://dicom.nema.org/). Fluoroscopic images of the knee and the patella that were captured during the whole knee lunging process were imported into the registration software (Figure 3). The technical details were also described in Appendix 2.

### 2.4 Data analysis

We achieved patellar tracking using patellar flexion/extension, varus/valgus, medial/lateral tilt, anteroposterior translation, medial/lateral translation, and proximal/distal translation. Each variable was described based on its mean and standard deviation at TFJ flexion angles of 0°, 30°, 60°, 90°, and 120°. In addition, we compared tibial rotations, varus/valgus, and the medial/lateral translation of the TFJ at the same flexion knee angles to observe whether any differences existed between knee joints with differing ACL and meniscus statuses. A repeated-measures ANOVA method was used to detect the effect of knee flexion on any variable. The Student-Newman-Keuls post-hoc test was used to detect significant differences between the groups. Significance was reported where *p* < 0.05. A post-hoc power analysis was performed to test the main significant results using the module Repeated measures analysis of PASS 15.0 (NCSS, LLC. Kaysville, UT, United States) (Naik and Rao, 2001). The means of patellar flexion angle of ACLI and Group 1–4 were 32.7, 25.9, 27.0 26.9 24.4°, respectively. The means of patellar flexion angle at TFJ flexion angles of 0°, 30°, 60°, 90°, and 120° were 5.5, 9.8, 19.2, 38.3, 64.0, respectively. The significance level was set to 0.05. The sample size per group were 15. The standard deviation was 10.1. Then, the statistical power was calculated to be 89.13% to detect significant differences between groups, indicating the sample size was enough for the significant results of patellar flexion angle. As for patellar medial/lateral tilt, the means of patellar lateral tilt angle of ACLI and Group 1–4 were 2.4, 6.1, 9.9, 8.0, and 7.7°, and the means at TFJ flexion angles of 0°, 30°, 60°, 90°, and 120° were 6.0, 6.1, 6.8, 6.8, and 8.4°. The standard deviation was 8.8. As for patellar lateral translation, the means of patellar lateral translation of ACLI and Group 1–4 were 5.1, 6.0, 7.9, 6.0, and 7.0 mm, and the means of patellar lateral translation at TFJ flexion angles of 0°, 30°, 60°, 90°, and 120° were 8.3, 5.2, 5.6, 6.2, and 6.5 mm. The standard deviation was 3.4. The results showed the statistical power for the remained two significant parameters were 91.36% for patellar lateral tilt and 90.25% for patellar lateral translation.

## 3 Results

### 3.1 Patellofemoral joint kinematics

#### 3.1.1 Patellar tilting

**FIGURE 4:**
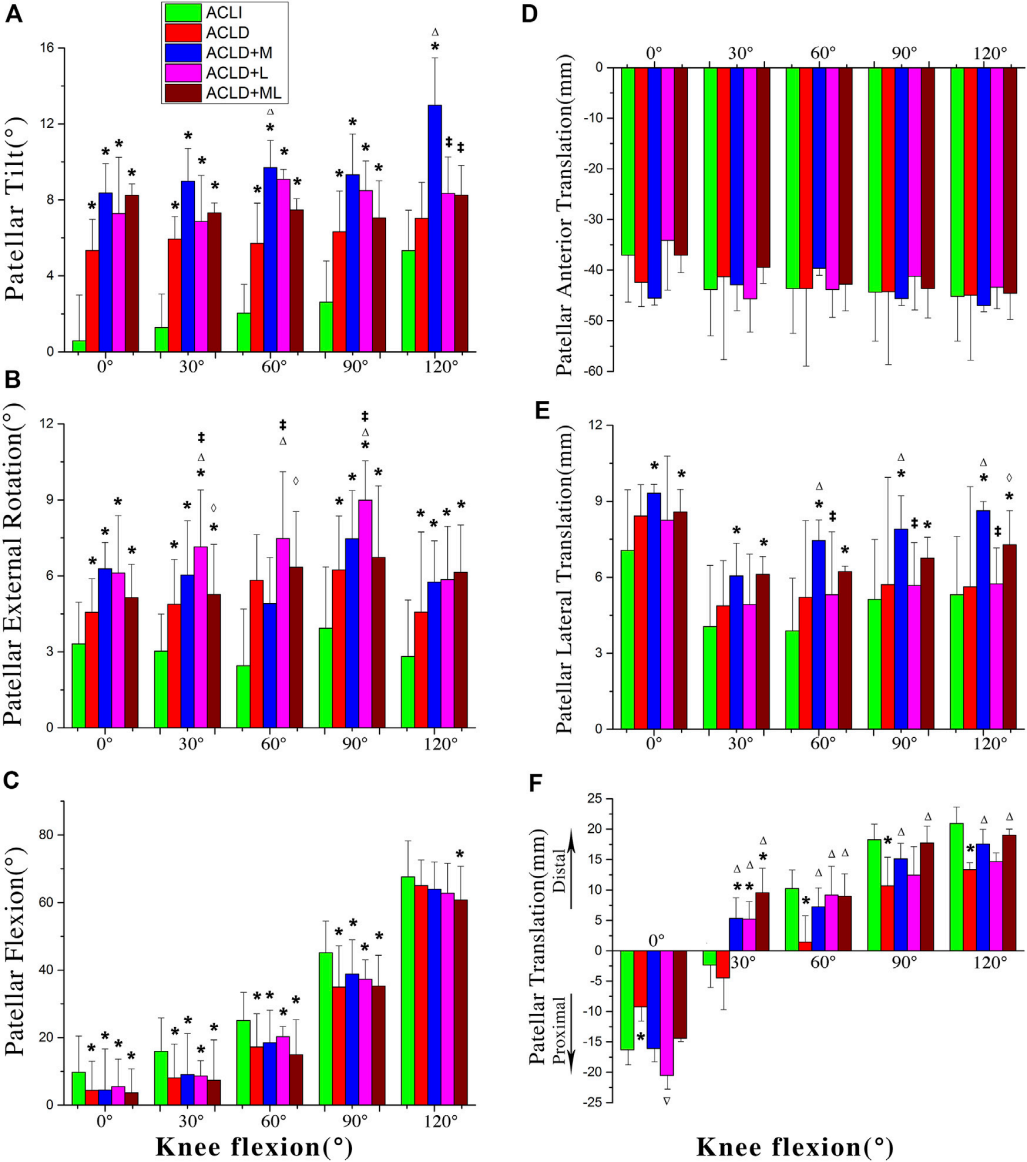
Motions of the patella with respect to the femur among five groups. ACLI = anterior cruciate ligament-intact knees (contralateral sides); ACL-D = ACL-deficient patients with isolated ACL deficiency (group I), combined ACL and medial meniscus injuries (group II), and combined ACL and lateral meniscus injuries (group III), combined ACL and medial-lateral meniscus injuries (group IV). Statistically significant difference (*p* < 0.05) compared with *intact, ^∆^group 1, ^‡^group 2, and ^⋄^group 3.

ACLD groups showed significant increased lateral tilt during almost the whole knee flexion procedure (Figure 4A). The increase was more significant in ACLD knees with medial meniscus deficiency than the other ACLD knees. At the terminal phase of the single-leg lunge (120° of knee flexion), only Group 2 showed a significant increase of patella lateral tilt compared to ACL-intact knees (12.9 ± 9.6° vs. 5.3 ± 8.2°, *p* = 0.002) and exhibited the highest lateral tilt. Groups 2 and 3 showed the highest lateral tilt (12.9 ± 9.6° and 9.1 ± 5.9°, respectively) at different flexion angles while groups 3 and 4 showed a steady pattern of lateral tilt during the whole flexion procedure. It is interesting to find that groups 1 and 4 showed consistent lateral tilt and exhibited few changes.

#### 3.1.2 Patellar internal/external rotation

In the frontal plane, these groups had similar kinematic profiles (Figure 4B). Generally, the patella showed consistently increased external rotation when the knee flexed. For internal/external rotation, ACLD knees exhibited an increase of about 3–5° in external rotation compared to ACLI knees (*p* < 0.05). Compared to the range of motion (ROM) the sagittal plane, the ROM in the frontal plane was smaller and approximately 6–8° throughout the single-leg lunge procedure. It was interesting to find that participants in Group 3 showed more external rotation than those with other types of ACLD knees.

#### 3.1.3 Patellar flexion

The predominant motion of the PFJ during the single-leg lunge occurred in the sagittal plane (Figure 4C). In the sagittal plane, the ACLD knees, with or without meniscal deficiency, had significantly less flexion than the ACLI knees (about 5–10°). For Group 4, in particular, this reduction in flexion was more significant than the reduction between ACLI knees and groups 1, 2, and 3 at 60° of knee flexion. In the ACLI group, the patellar flexion angle was found to be about half of knee flexion.

#### 3.1.4 Anteroposterior translation

In general, the patellar showed anterior patellar translation during knee flexion (Figure 4D). No significant difference was found among the groups through the whole measured range, even though the isolated ACLD group and ACLD groups with medial meniscus deficiencies showed more anterior translation than those with ACLI knees in partial phases of the single-leg lunge procedure.

#### 3.1.5 Mediolateral translation

ACLD groups with medial meniscal deficiencies showed consistent increased lateral patellar translations (about 2–4 mm) compared to the ACLI group during the single-leg lunge procedure (Figure 4E). No significant differences were found between the ACLI group and the ACL isolated deficient group. Similar findings were shown between the ACLI group and the ACLD groups with concomitant medial and lateral meniscus deficiencies (*p* < 0.05) at different flexion angles.

#### 3.1.6 Proximal/distal translation

The patella exhibited a distinguished movement pattern concerning proximal-distal translation (Figure 4F). In ACL-intact knees, initially, the center of patella was proximal to the femur origin located at the center of the femur condyle when the knee extended, up to 16.3 ± 9.2 mm. The patella moved distally and was distal to the femur as the knee flexed, up to 20.9 ± 13.3 mm. A significant reduction of proximal translation (about 7 mm) was identified in the ACL isolated deficient group compared to the ACLI group (*p* = 0.029) when the knee extended. As the knee flexed, a significant reduction of distal translation (about 7 mm) was shown in the ACL isolated deficient group at 60°, 90°, and 120° of knee flexion. No significant proximal-distal translation was detected between the ACLI group and ACL non-isolated deficient groups, even though the latter groups showed less distal patellar translation of about 3–5 mm.

### 3.2 Tibiofemoral joint kinematics

**FIGURE 5:**
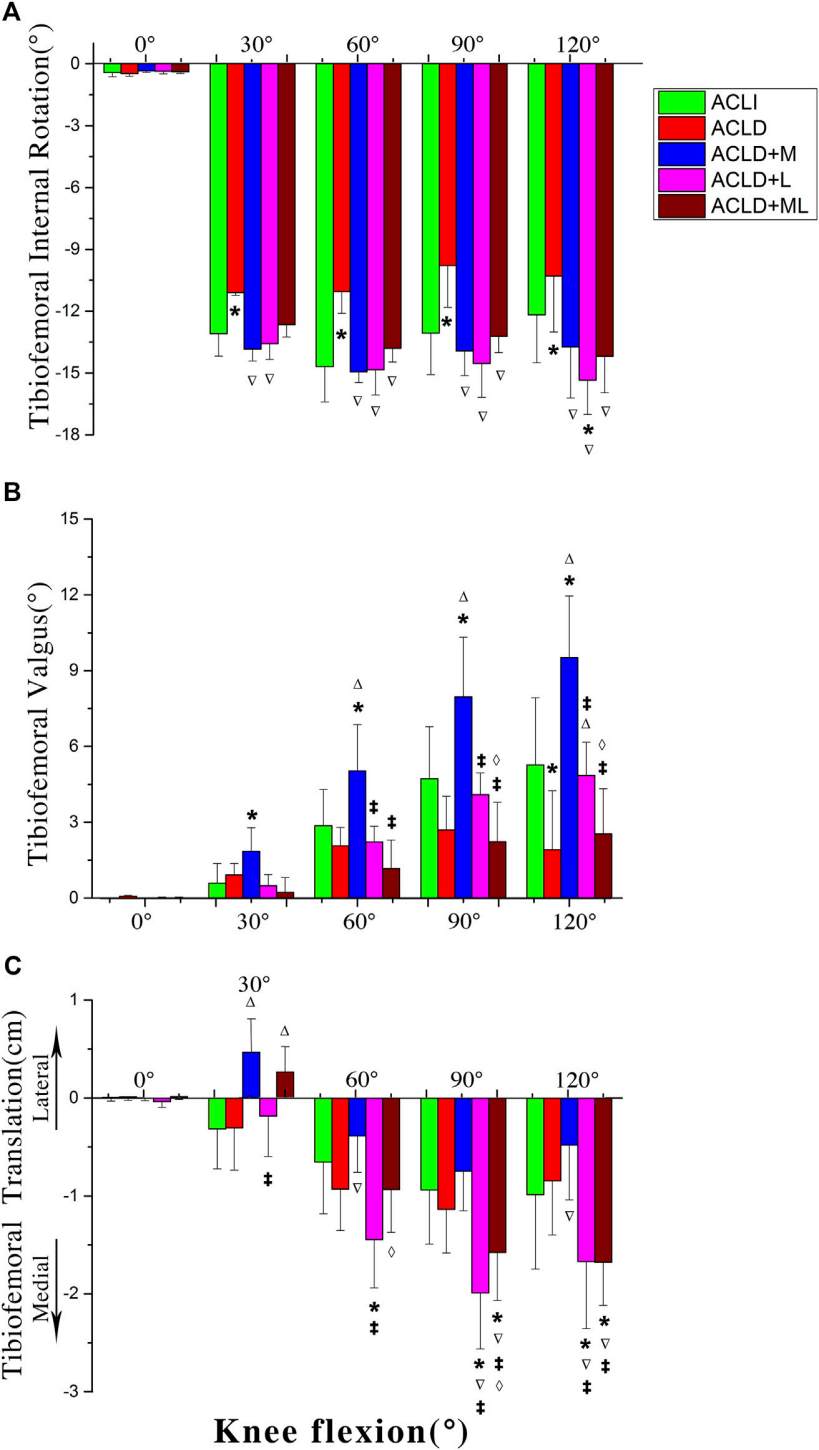
Motions of the tibia with respect to the femur among five groups. ACLI = anterior cruciate ligament-intact knees (contralateral sides); ACL-D = ACL-deficient patients with isolated ACL deficiency (group I), combined ACL and medial meniscus injuries (group II), and combined ACL and lateral meniscus injuries (group III), combined ACL and medial-lateral meniscus injuries (group IV). Statistically significant difference (*p* < 0.05) compared with *intact, ^∆^group 1, ^‡^group 2, and ^⋄^group 3.

We compared TFJ rotations between ACL-intact knees and ACLD knees with and without concomitant meniscus deficiency at 0°, 30°, 60°, 90°, and 120° of knee flexion. For knee rotations, both ACLI knees and ACLD knees showed internal tibial rotations during single-leg lunges (Figure 5A). The isolated ACLD knees showed significantly reduced internal tibial rotations (about 2–3°) when compared to ACLI knees. No significant difference was found between ACLI knees and ACLD knees with concomitant meniscus deficiency.

#### 3.2.1 Knee varus/valgus

For knee varus/valgus, both ACLI knees and ACLD knees showed valgus during single-leg lunges (Figure 5B). The ACLD knees with concomitant medial meniscus deficiency showed significantly increased valgus (about 2–5°) compared to other groups. No significant difference was found between ACLI knees and ACLD knees with or without concomitant meniscus deficiency, except the comparison between ACLI knees and ACLD knees with concomitant medial meniscus deficiency.

#### 3.2.2 Knee mediolateral translations

The knee joint exhibited a single movement pattern regarding mediolateral translation (Figure 5C). The tibia showed medial tibial translations during almost the whole procedure of a single-leg lunge. The ROM in mediolateral translation was small (1–2 cm). The ACLD knees with concomitant lateral meniscus deficiency showed significantly increased medial tibia translations (about 10 mm) compared to other groups from 60° of knee flexion. The ACLD knees with concomitant medial and lateral meniscus deficiency also showed significantly increased medial tibia translations (about 10 mm) compared to other groups at terminal knee flexion ranges (90–120°).

## 4 Discussion

The present study determined the 6DOF PFJ kinematics of ACLD knees with and without meniscal tears during a single-leg lunge. In the present study, significant statistical kinematic differences were detected in both PFJ rotation and translation between groups. The results of the present study support our initial hypothesis that the patellofemoral kinematic response of ACLD knees could be significantly affected by the presence and type of meniscal tears. The findings indicate that rehabilitation with CKC quadriceps exercises, such as single-leg lunges, led to significantly altered PFJ kinematics in ACLD knees when compared to those of ACLI knees. Our data also suggests that further research on different surgical approaches to the restoration of PFJ kinematics when ACL deficiency is combined with meniscal injuries may be necessary.

In recent years, many researchers have reported that the prevalence of PFJ OA after ACL injury has been increasing (Culvenor et al., 2013; Friel and Chu, 2013; Huang et al., 2020). The relationship between general PFJ OA and the alteration of PFJ contact remains unknown. It is well established that OA may be initiated following ACL transection because of an overloading of specific regions of the joint, either because of the altered contact mechanics or disrupted joint kinematics, despite a general decrease in the contact pressure. Interpatient kinematics in ACLD patients with or without meniscus tears are widely investigated based on the determinations of kinematics (Hosseini et al., 2014; Zhang et al., 2016). However, most of these studies were investigations of the effects of ACL and meniscus status on the TFJ rather than the PFJ.

Doing lunges is an effective exercise for strengthening, sculpting, and building several muscles and muscle groups, including the quadriceps (or thighs), the gluteus maximus (or buttocks), and the hamstrings. It is often used by athletes in cross-training for sports, by weight-trainers as a fitness exercise, and by practitioners of yoga as part of an asana regimen. Morrissey et al. reported that CKC exercises, such as lunges, are relatively safe for the PFJ, but they did not specify the status of the participants’ meniscuses (Morrissey et al., 2002). The kinematic changes that occur during single-leg lunges after an ACLD, with or without meniscal tear, may have significant effects on the stability of the PFJ, which may result in joint instability and cartilage degeneration. Regarding specific changes between the ACLD and ACLI groups, significant kinematic changes were detected in the transverse plane. In the transverse plane, ACLD groups showed significant increased lateral tilt during almost the whole knee flexion process. The increase was more significant in ACLD knees with medial meniscus deficiency than the other ACLD knees. From an anatomical viewpoint, the ACL has an oblique medial orientation from the femur to the tibia. With a ACL tear, the femur exhibits external rotation. The increased femoral external rotation indicates the patients’ tendency to avoid unstable knee positions because of the loss of passive constraint associated with a complete rupture of the ACL, or to avoid loading the injured ACL if it was partially torn. With increased femoral external rotation, due to the distal end of the quadriceps tendon attached on the superior ridge of the patella, the patella showed increased lateral tilt. There is a high possibility that these changes will result in changes to the compression of cartilage on the lateral compartment of the PFJ. A systematic review has shown that in ACLD knees, the medial compartment of the PFJ is vulnerable to cartilage degeneration and osteoarthritis development (van Meer et al., 2015). Moreover, Wang et al. reported that partial medial meniscectomy (<33% of medial meniscus resection) led to prevalent PFJ OA (OR, 13.76; 95% CI, 1.52–124.80) (Wang et al., 2012). They speculated that when coupled with quadriceps weakness seen in medial meniscectomy, a varus alignment might increase the stress on the medial PFJ, consequentially increasing the risk of PFJ OA development. These findings indicate that the meniscus maintains PFJ contact mechanics. Thus, the ACLD groups exhibited significantly more lateral tilt than those with ACLI knees. Interestingly, the increased lateral tilt was more significant in ACLD knees with medial meniscus deficiency than the other ACLD knees. The medial meniscus is often injured when the knee is twisted or sprained with sudden force. It is less mobile than the lateral meniscus because it is firmly attached to the tibial collateral ligament. External femoral rotation puts the most strain on the meniscus while femoral internal rotation is the least strenuous. A medial meniscus deficiency indicates that femoral external rotation was hindered when the ACL ruptured, leading to a significant increase in lateral tilt.

Regarding the frontal plane, in general, patellar rotation in the frontal plane in the patients of the four groups exhibited similar patterns. In particular, isolated ACLD knees showed more significant increases in external rotation (about 3–5°) than other ACLD knees. As mentioned above, with a ruptured ACL, the femur exhibits external rotation. Hence, the patella showed increased external rotation because the distal attachment of the quadriceps tendon is attached on the femur. The magnitudes of increased external rotation in concomitant meniscus-deficient groups were smaller than those in the isolated ACLD group. Due to the altered kinematics, the upper ridge on the back surface of the patella moved closer on the lateral compartment of the femoral trochlear groove. Likewise, the lower ridge moved closer to the medial compartment of the femoral trochlear groove. As a result, the alterations of contact locations are likely to lead to compress between the patella and the femoral trochlear groove.

Because the patellar tendon attaches the tibial tubercle, the movement of the tibia is believed to affect patellar movements. The isolated ACLD knees showed significantly reduced internal tibial rotations (equivalent to external femoral rotation, about 2–3°) compared to ACL-intact knees. Hence, we found that the patella demonstrates the most significant lateral tilting in isolated ACLD. The ACLD knees with concomitant medial meniscus deficiency showed significantly increased valgus (about 2–5°) compared to those of in other groups. We speculated that due to the increased knee valgus angle, the patella tends to rotate externally. The TFJ kinematics presented in the current study are consistent with those reported in previous studies (Van de Velde et al., 2008; Zhang et al., 2016). The reasons for which the TFJ kinematics were altered in this way were discussed in these studies. The ACLD knees with concomitant lateral meniscus deficiency showed significantly increased medial tibia translations compared to other groups at 60° of knee flexion. The ACLD knees with combined medial and lateral meniscus deficiency also showed significantly increased medial tibia translations compared to other groups at terminal knee flexion ranges (90–120°). These kinematic changes in the TFJ are likely to lead to PFJ kinematic changes, resulting in abnormal contact locations between the patella and the femur, thus leading to the onset of adverse PFJ outcomes. Regarding the analysis on other kinematic alterations of the PFJ and the TFJ, it was shown in Appendix 3.

There are some potential limits in the present study. Firstly, the absence of a pressure sensor or force plate to record the PFJ force moment made the measurement less straightforward. The kinematics are not fully representative of the contact mechanics in the PFJ. Second, only 60 subjects were enrolled in the current study. The relatively small sample size may not be enough to represent the general characteristics of different patterns of meniscal tears in ACLD patients. Third, the patients in the test were generally young and are not representative of all patients. Therefore, the results may not be consistent to old populations. However, it is important to note that the age of the patients in the present study was closely consistent with the demographics of the population most commonly treated for ACLD knees. Fourth, intragroup Q angle variations between both legs of a certain patient, intragroup mechanical axis variations, the intragroup female/male ratio, and sex-related bony anatomy differences were not considered within the design of the study. Fifth, we briefly discussed the timing of differences observed relative to portions of the lunge procedure at which the ACL is loaded. This might not fully explain when, during the single-leg lunge procedure, the meniscus would be expected to play a greater role than the ACL.

## 5 Conclusion

The present study showed that a combined ACL/meniscal injury changes the ACLD knees’ PFJ kinematics, depending on the location of the meniscal injuries during a single-leg lung exercise. Based the effects of meniscal tears on the PFJ kinematics, target treatments for patients with combined ACL and meniscal tears should be proposed to protect the PFJ joint from abnormal kinematics and subsequent postoperative cartilage degeneration in some CKC exercise programs, such as single-leg lunge.

## Data Availability

The data are available from the corresponding authors upon a reasonable request.
